# Reproductive Hormone-Dependent and -Independent Contributions to Developmental Changes in Kisspeptin in GnRH-Deficient Hypogonadal Mice

**DOI:** 10.1371/journal.pone.0011911

**Published:** 2010-07-30

**Authors:** John C. Gill, Oulu Wang, Shelley Kakar, Enzo Martinelli, Rona S. Carroll, Ursula B. Kaiser

**Affiliations:** 1 Division of Endocrinology, Diabetes, and Hypertension, Brigham and Women's Hospital, Harvard Medical School, Boston, Massachusetts, United States of America; 2 Harvard Reproductive Endocrine Sciences Center, Boston, Massachusetts, United States of America; 3 Division of Endocrinology, Children's Hospital Boston, Boston, Massachusetts, United States of America; University of Córdoba, Spain

## Abstract

Kisspeptin is a potent activator of GnRH-induced gonadotropin secretion and is a proposed central regulator of pubertal onset. In mice, there is a neuroanatomical separation of two discrete kisspeptin neuronal populations, which are sexually dimorphic and are believed to make distinct contributions to reproductive physiology. Within these kisspeptin neuron populations, *Kiss1* expression is directly regulated by sex hormones, thereby confounding the roles of sex differences and early activational events that drive the establishment of kisspeptin neurons. In order to better understand sex steroid hormone-dependent and -independent effects on the maturation of kisspeptin neurons, hypogonadal (*hpg*) mice deficient in GnRH and its downstream effectors were used to determine changes in the developmental kisspeptin expression. In *hpg* mice, sex differences in Kiss1 mRNA levels and kisspeptin immunoreactivity, typically present at 30 days of age, were absent in the anteroventral periventricular nucleus (AVPV). Although immunoreactive kisspeptin increased from 10 to 30 days of age to levels intermediate between wild type (WT) females and males, corresponding increases in Kiss1 mRNA were not detected. In contrast, the *hpg* arcuate nucleus (ARC) demonstrated a 10-fold increase in Kiss1 mRNA between 10 and 30 days in both females and males, suggesting that the ARC is a significant center for sex steroid-independent pubertal kisspeptin expression. Interestingly, the normal positive feedback response of AVPV kisspeptin neurons to estrogen observed in WT mice was lost in *hpg* females, suggesting that exposure to reproductive hormones during development may contribute to the establishment of the ovulatory gonadotropin surge mechanism. Overall, these studies suggest that the onset of pubertal kisspeptin expression is not dependent on reproductive hormones, but that gonadal sex steroids critically shape the hypothalamic kisspeptin neuronal subpopulations to make distinct contributions to the activation and control of the reproductive hormone cascade at the time of puberty.

## Introduction

Pubertal maturation and reproductive function rely on the integration of environmental and physiological cues by the GnRH neuronal network [1]. GnRH release from these neurons is orchestrated by neurotransmitters, neuropeptides and hormones during the activation and subsequent maintenance of reproductive function [2]. Overwhelming evidence in mammalian species has shown that through its receptor (Kiss1R) located on GnRH neurons, the neuropeptide kisspeptin is a direct component of the reproductive control system and is a potent stimulator of GnRH release [3]. Kisspeptinergic activity satisfies important criteria as a signal necessary for puberty: 1) disruptive mutations and genetic models with ablated kisspeptin signaling are hypogonadal and fail to undergo pubertal maturation [4], [5]; 2) at puberty, increased hypothalamic kisspeptin corresponds with the time of increased GnRH release [6], [7]; and 3) kisspeptin administration to juvenile animal models elicits precocious activation of the reproductive axis [8]. Currently, it remains to be definitively determined whether the kisspeptin system is the driver of hypothalamic-pituitary-gonadal (HPG) axis maturation, or rather is dictated, in part or whole, in response to this axis. Importantly, the influence of the HPG axis itself on the sexual differentiation of hypothalamic kisspeptin organization and function has not been fully elucidated.

In adult mice, kisspeptin neurons are predominantly localized in two brain regions, each differentially regulated by gonadal steroids acting through ERα and likely AR [9], [10]. First, the arcuate nucleus (ARC) population of kisspeptin neurons has higher levels of *Kiss1* expression under conditions of low circulating gonadal steroids. In the ARC, GnRH neuronal processes near the median eminence, where GnRH is released, are associated with kisspeptin immunoreactive fibers [11]. Under conditions of high levels of circulating gonadal steroids, ARC *Kiss1* expression decreases [10], thus ARC kisspeptin neurons are predicted to play a functional role in negative feedback regulation. Distinct from the kisspeptin neurons in the ARC, a discrete, second kisspeptin population is localized in the anteroventral periventricular nucleus (AVPV). This population of kisspeptin neurons is sexually dimorphic, with 10–20 fold more kisspeptin-positive neurons in females than males [7], [12]. In contrast to the ARC, AVPV *Kiss1* expression increases in response to gonadal steroids [10] and AVPV *Kiss1* expression has also been shown to vary during the rodent estrus cycle, culminating with the highest levels of expression coincident with the ovulatory LH surge [9], [13], [14]. These findings provide a strong argument for a role for the AVPV kisspeptin neurons as effectors of the estrogen positive feedback mechanism leading to the gonadotropin surge in females.

Ultimately, evidence supports actions of kisspeptin upstream of GnRH in the neuroendocrine regulation of reproduction. In the absence of GnRH, reproductive maturation does not occur, although it might be predicted that central activation of the neuronal network upstream of GnRH would proceed normally as long as these stimuli are not dependent on reproductive hormones. The hypogonadal *(hpg)* mouse has a large deletion in the *Gnrh1* gene, resulting in failure to synthesize GnRH [15]. As a result, *hpg* homozygous mice have a phenotype characterized by low gonadotropin levels (LH and FSH) and undeveloped reproductive tracts in both males (infantile sizes of testes and seminal vesicle) and females (small, immature ovaries and thread-like uterus), indicating the presence of minimal, if any, gonadal steroid hormones [16]. Accordingly, the *hpg* mouse provides an excellent model in which to study the onset and maturation of kisspeptin expression, without confounding developmental and trophic actions of gonadal sex steroids and other reproductive hormones.

In this study, we have measured developmental and sex-based differences in hypothalamic kisspeptin expression and distribution in the *hpg* mouse model, to address questions of the programming role of the HPG axis itself on the kisspeptinergic system. Our results indicate that the developmental changes and sex-based differences in the hypothalamic kisspeptin neuronal network are influenced by both reproductive hormone-dependent and -independent mechanisms, and suggest that the ARC kisspeptin neuronal subpopulation plays an important role in the central activation of the reproductive hormone cascade necessary to initiate puberty. Furthermore, loss of the normal positive feedback response of AVPV kisspeptin neurons to estrogen in *hpg* mice suggests that exposure to reproductive hormones during development may contribute to the establishment of the ovulatory gonadotropin surge mechanism.

## Results

### Absence of Sexual Dimorphism in the AVPV Kisspeptin Neurons of *hpg* Mice

**Figure 1:**
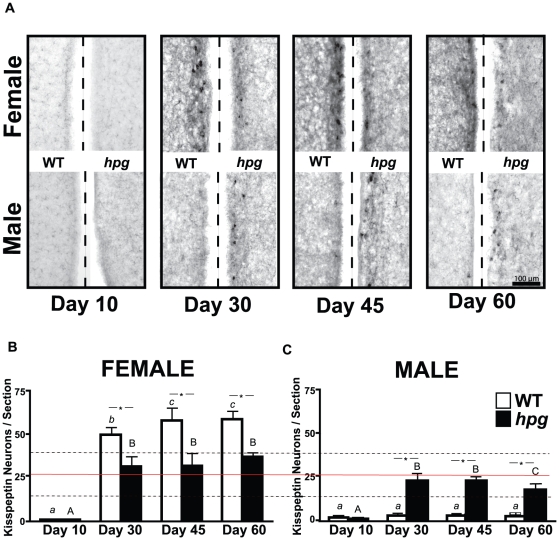
Immunocytochemistry (ICC) of kisspeptin neurons in the AVPV during postnatal development in wild-type (WT) and *hpg* mice. **A**) Representative images of kisspeptin immunostaining from AVPV regions along the third ventricle (*dashed line*) of female and male WT and *hpg* mice at postnatal ages 10, 30, 45 and 60 days. **B, C**) Quantitation of the number of immunoreactive kisspeptin neurons/section in the AVPV of WT and *hpg* female (**B**) and male (**C**) mice. Values are mean ± SEM. WT, *white bars*; *hpg*, *black bars*. Significant differences between ages are denoted by different letters (WT, *lower case*, *hpg*, *upper case*; ANOVA, Neuman-Keuls post hoc); *, denotes significant difference between genotypes. *Solid horizontal line* across B and C indicates the mean of the number of immunoreactive neurons in the AVPV of *hpg* females and males from P30 onward, and the *dotted lines* indicate ±2 standard deviations from the mean. P<0.05. Scale bar  = 100 µm.

To determine the impact of reproductive hormones on kisspeptin neurons in females and males across postnatal maturation, kisspeptin-positive neurons were compared in coronal brain sections of WT and *hpg* mice at 10, 30, 45 and 60 days of age. Kisspeptin neurons were identified by immunocytochemistry (ICC) in WT mice in both the AVPV and periventricular nucleus (PEN) in the immediate area surrounding the third ventricle, in a rostral-caudal continuum, recently described as the RP3V [17]. Consistent with previous reports [7], the number of kisspeptin-immunoreactive neuronal cell bodies in the AVPV was sexually dimorphic with WT female mice possessing 10–20 fold more kisspeptin-positive neurons than WT males. Only at the earliest developmental age examined, 10 days, was the number of AVPV kisspeptin-positive neurons similar in WT females (0.6±0.2 neurons/section) and males (1.5±0.7) (**Fig. 1**). As expected, the number of immunoreactive kisspeptin neurons in the AVPV increased significantly in female WT mice during postnatal development (P<0.0001; ANOVA), and during the pubertal transition between postnatal day (P) 10 and P30 kisspeptin neurons increased to 49.6±4.7 neurons/section (P<0.05; Neuman-Keuls post-hoc). In WT males, despite the increase in staining intensity of immunoreactive kisspeptin fibers and a small increase in the number of kisspeptin-immunoreactive neurons with age (2.8±0.9 neurons/section at P45; **Fig. 1A, B**), this increase did not reach significance (P>0.05 vs. P10; Mann-Whitney), in contrast to a previous ICC study in WT mice [7]. Nonetheless, this presence of kisspeptin neurons in the AVPV of WT male mice marked a distinction from the absence of immunoreactive kisspeptin in the AVPV of the male rat reported recently by Bentsen, *et al.* [18].

In *hpg* mice at P10, few kisspeptin-positive neurons were found in the AVPV of either sex (females, 0.4±0.2 neurons/section; males, 0.6±0.6 neurons/section) and were equivalent to WT mice at this age. During postnatal development, female *hpg* mice demonstrated a significant increase in the number of kisspeptin-immunoreactive neurons, despite the absence of reproductive hormones (P<0.05; ANOVA). Interestingly, there was also a significant increase in male *hpg* AVPV kisspeptin-positive neurons during maturation (P<0.05; ANOVA) **(** **Fig. 1** **)**. Moreover, the sexually dimorphic pattern observed in the AVPV of WT mice was no longer evident in *hpg* females (31.5±7.7 neurons/section) and males (22.4±2.9 neurons/section; P>0.05; t test) at P30 **(** **Fig. 1** **)**. By P30, the developmental increase in kisspeptin-positive neurons in the AVPV of *hpg* female mice was only half of that measured in WT females. In contrast, the increase in AVPV kisspeptin-positive neurons at P30 was 10-fold greater in *hpg* male mice than that observed in WT males at the same age. Comparisons of ages P30 onward by ANOVA showed no statistical difference between the numbers of kisspeptin-positive neurons in the AVPV of *hpg* females and males (P>0.05). Therefore, under the low reproductive hormone levels of *hpg* mice, immunostaining revealed marked alterations to the maturation of both female and male AVPV kisspeptin populations.

### Loss of Sexual Dimorphism in AVPV Kiss1 mRNA Levels in *hpg* Females and Males

**Figure 2:**
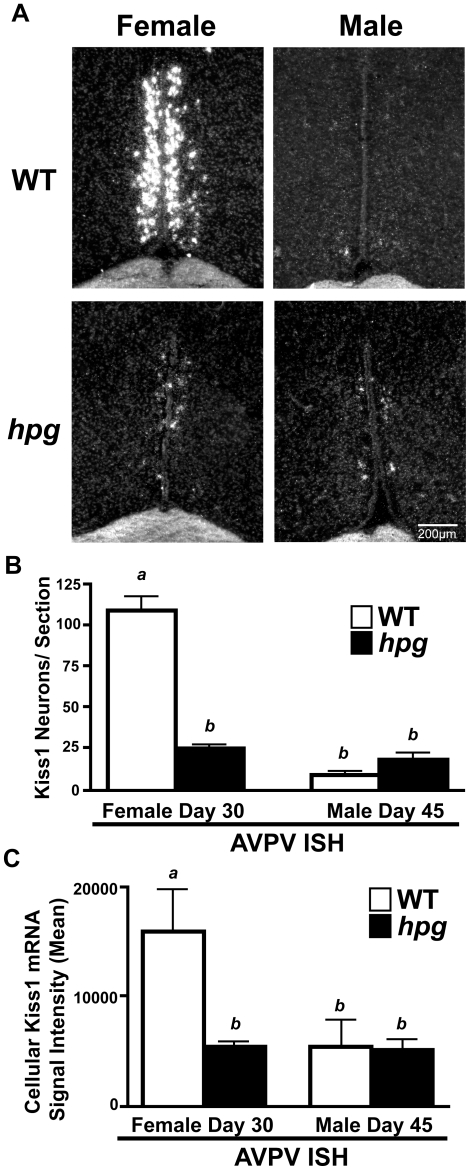
Comparison of *Kiss1* expression in the AVPV of WT and *hpg* mice. **A**) Representative dark-field images of silver grain Kiss1 mRNA signal as measured by in situ hybridization in the AVPV of pubertal-aged WT and *hpg* females (Day 30) and males (Day 45). **B**) Quantitation of the number of neurons positive for Kiss1 mRNA per section in the AVPV. **C**) Representation of average Kiss1 silver-grain signal per neuron by measurement of sum pixel brightness within identified neurons/section. Values are mean ± SEM. WT, *white bars*; *hpg, black bars*. Different letters denote significant differences (ANOVA, Neuman-Keuls post hoc), P<0.05. Scale bar  = 200 µm.

To determine whether the observed differences in kisspeptin immunostaining in the AVPV of WT and *hpg* mice are associated with changes in Kiss1 mRNA expression, in situ hybridization (ISH) was performed to compare WT and *hpg* mice at approximate ages of puberty, P30 in females and males at age P45 [7], [19]. Silver grain clusters, representing cells expressing Kiss1 mRNA, were found in the same hypothalamic locations as immunostained kisspeptin neurons **(** **Fig. 2A** **)**. In the AVPV of WT females and males, Kiss1 ISH demonstrated the same sexually dimorphic pattern observed by immunostaining, with more AVPV Kiss1 mRNA-positive neurons in females (109.0±8.6 neurons/section) than in males (9.2±1.7 neurons/section; P<0.05) **(** **Fig. 2B** **)**, values similar to previous studies [9], [10]. The number of neurons positive for Kiss1 mRNA in the AVPV of female *hpg* mice was significantly lower than in WT females (24.7±1.2 neurons/section; P<0.001; Neuman-Keuls post-hoc), whereas the number in the *hpg* males (17.1±2.9 neurons/section) showed a trend towards an increase compared to WT males but did not reach statistical significance (P = 0.057; Mann-Whitney) **(** **Fig. 2B** **)**. Most striking was the loss of sexual dimorphism, with no significant difference between the number of neurons positive for Kiss1 mRNA between the AVPV of *hpg* females and males. This finding correlated closely with the loss of sexual dimorphism observed by kisspeptin ICC. Image analysis software was used to estimate Kiss1 mRNA content per neuron by the level of silver grain signal brightness and demonstrated Kiss1 mRNA per neuron was reduced in *hpg* females compared to WT to further illustrate the loss of sexual dimorphism between female and male *hpg* mice **(** **Fig. 2C** **)**.

### Changes in AVPV *Kiss1* Gene Expression During Postnatal Development

**Figure 3:**
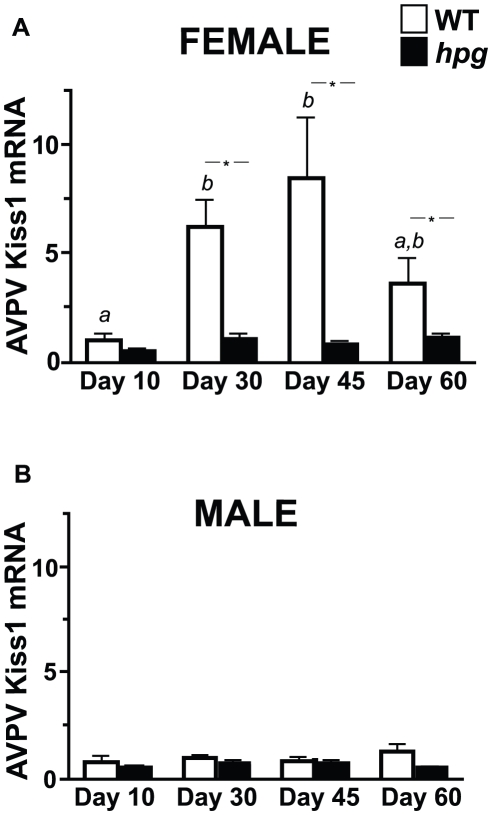
*Kiss1* expression in the AVPV of WT and *hpg* females and males across postnatal development. Quantitative analysis of Kiss1 mRNA levels in the AVPV of (**A**) females and (**B**) males as measured by qRT-PCR. Values are mean ± SEM. WT, *white bars*; *hpg, black bars*. Different letters denote significant differences (ANOVA, Neuman-Keuls post hoc); *, denotes significant difference between genotypes, P<0.05.

To next define the changes in *Kiss1* gene expression in the AVPV during postnatal development in a hypogonadal background, RNA was collected from the AVPV of female and male WT and *hpg* mice at ages 10, 30, 45 and 60 days to analyze Kiss1 mRNA expression by real-time qRT-PCR (**Fig. 3**). All Kiss1 mRNA values were calculated relative to the P10 female AVPV values (mean Ct). Comparing WT females and males, AVPV Kiss1 mRNA levels were similar in prepubertal mice at P10 (1.41±0.70, N = 5, and 0.73±0.31, N = 4, respectively). However, Kiss1 mRNA levels were significantly higher in the WT females than males by P30, and remained higher than in the males at P45 and P60 (P<0.05), typifying the known sexually dimorphic pattern of *Kiss1* expression in the AVPV. In contrast, the difference between sexes was not detected in *hpg* mice, consistent with our observations by ISH and ICC, further corroborating the loss of sexual dimorphism of kisspeptin expression in the AVPV of *hpg* mice.

During pubertal development, between P10 and P30, AVPV Kiss1 mRNA levels increased 4-fold in WT females (P10, 1.41±0.70; P30, 6.24±1.24, N = 5; P<0.05; Neuman-Keuls post-hoc), indicating a developmental increase in *Kiss1* expression consistent with the increase in kisspeptin neurons detected by ICC **(** **Fig. 3A** **)**. In contrast, in the *hpg* females, no significant increases in Kiss1 mRNA levels with age were detected. In both WT and *hpg* males, AVPV Kiss1 mRNA levels were low and did not increase during postnatal maturation (ages P10 to P60) **(** **Fig. 3B** **)**. In contrast to the ICC results measuring kisspeptin immunoreactive neurons, the developmental increase in Kiss1 mRNA levels was not detected by qRT-PCR in the AVPV of *hpg* males. However, these data were consistent with the Kiss1 ISH analysis that only demonstrated a difference in *Kiss1* expression in the AVPV of WT and *hpg* females but no difference between WT and *hpg* males at pubertal ages.

### Kisspeptin Immunostaining Reveals an Altered Distribution Pattern in the ARC of *hpg* Mice

**Figure 4:**
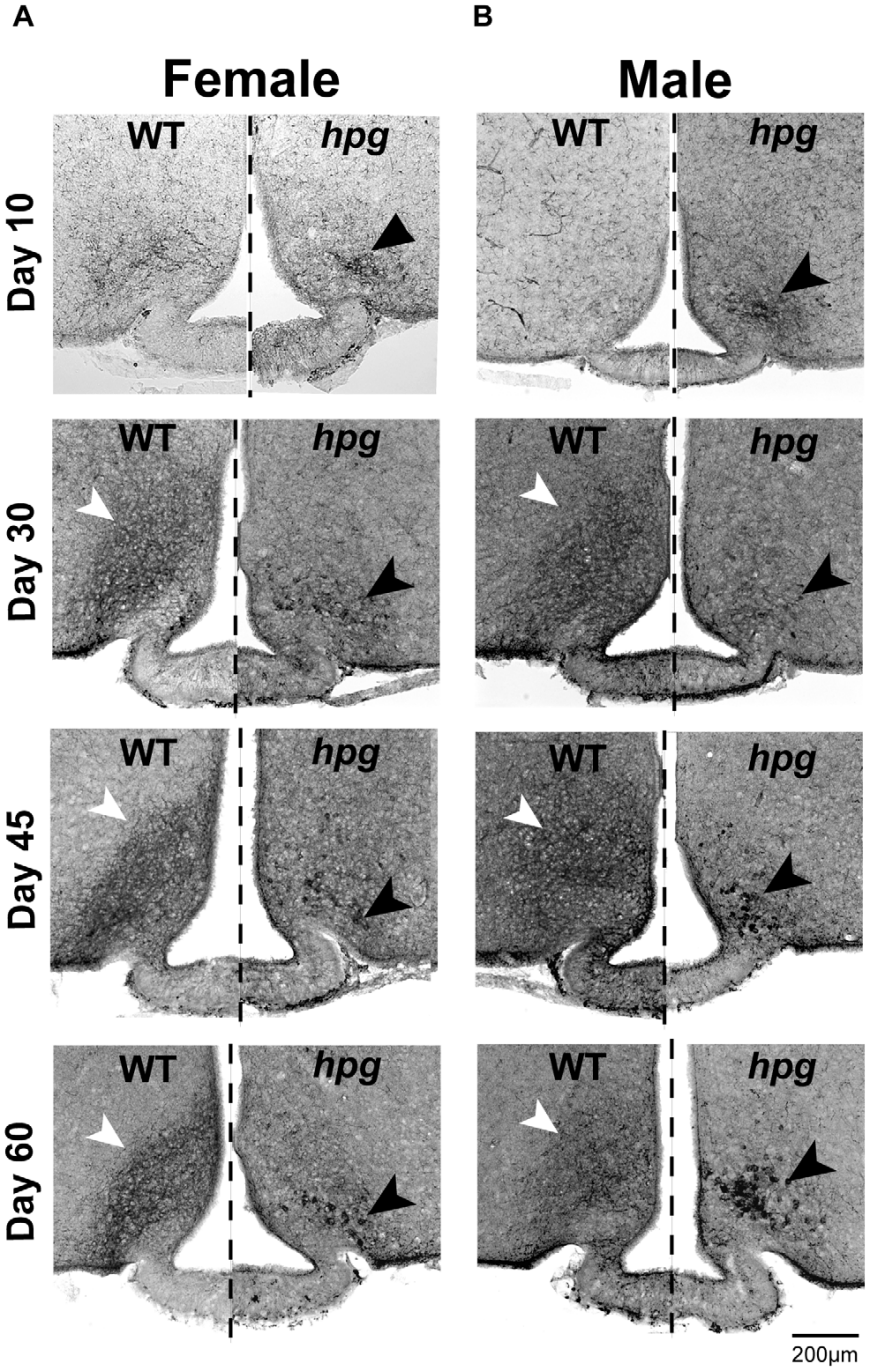
Altered distribution pattern of kisspeptin immunostaining in the ARC of *hpg* mice. Kisspeptin ICC across postnatal development (age 10, 30, 45 and 60 days) in WT and *hpg* females (**A**) and males (**B**). Kisspeptin ICC staining pattern in the ARC of both female and male WT mice (*left of dashed line*) is dominated by densely stained fibers that obscure kisspeptin-positive cell bodies (*white arrowheads*). In *hpg* mice (*right of dashed line*), ARC kisspeptin ICC shows reduced fiber staining and increased clusters of large, darkly stained cell bodies (*black arrowheads*). Scale bar  = 200 µm.

The second population of neurons immunoreactive for kisspeptin, in the ARC, localized more caudally and separate from the AVPV [7], was stained by ICC in female and male WT and *hpg* mice. ARC kisspeptin immunoreactivity was predominated by a pattern of densely stained fibers comprising a thick neuropil immediately dorso-lateral to the area surrounding the median eminence **(** **Fig. 4** **)**. WT kisspeptin immunoreactive cell bodies were difficult to distinguish from the underlying pattern of neuropil fiber staining in the ARC and so could not be counted. The immunoreactive density of the fiber staining for kisspeptin increased qualitatively in the ARC in both female and male WT mice during development, peaking at P45. In *hpg* mice, however, ARC kisspeptin immunostaining patterns were markedly different from WT at all ages in two major ways (**Fig. 4**). First, there were notable reductions in the neuropil immunoreactivity resulting in reduced kisspeptin fiber staining. Second, conspicuous clusters of large, darkly stained immunoreactive kisspeptin cell bodies, not present in the WT ARC, predominated the ventral and lateral aspects of the *hpg* ARC. Kisspeptin staining showed no appreciable differences between male and female ARC kisspeptin immunoreactivity for either genotype.

### Increased *Kiss1* Expression in the ARC of *hpg* Mice

**Figure 5:**
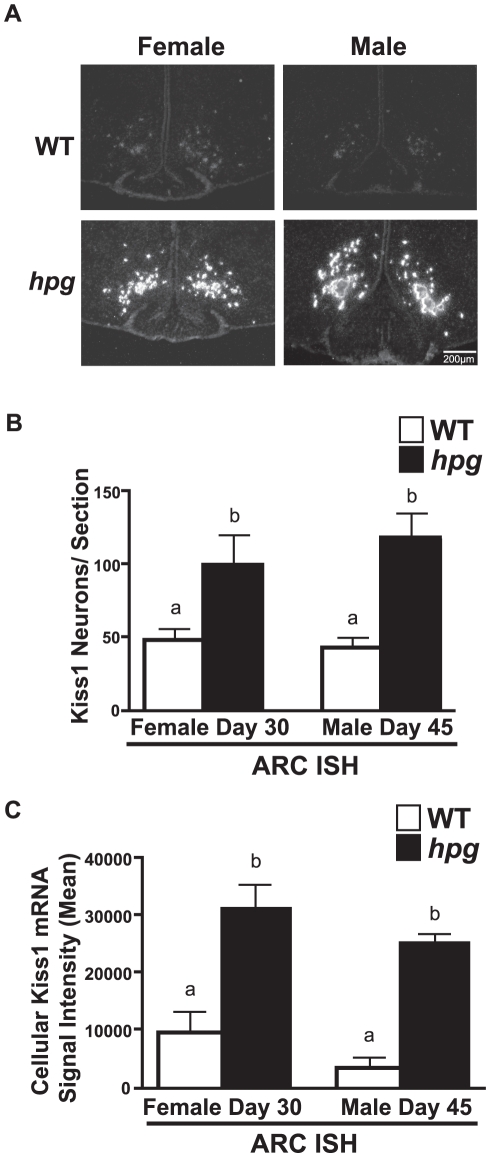
*Kiss1* expression is increased in the ARC of female and male *hpg* mice. **A**) Representative dark-field images of silver grain Kiss1 mRNA signal as measured by ISH in the ARC of pubertal-aged WT and *hpg* females (30 days) and males (45 days). Scale bar  = 200 µm. **B**) Male and female Kiss1 positive neurons/section in the *hpg* ARC compared to WT. **C**) Mean of representative Kiss1 mRNA silver grain signal per neuron in the male and female *hpg* ARC sections compared to WT (mean signal intensity). Values are mean ± SEM. WT, *white bars*; *hpg, black bars*. Different letters denote significant differences, P<0.05.

Since the levels of kisspeptin staining in the ARC were difficult to quantify by ICC, ISH was used to determine if the differences in ARC kisspeptin immunostaining patterns between the WT and *hpg* mice were associated with differences in *Kiss1* gene expression. In both females and males, a robust increase in silver grain signal density was observed in the ARC of *hpg* mice when compared to WT **(** **Fig. 5** **)**. There were no sex differences in ARC Kiss1 mRNA-positive neurons between mice of the same genotype. However, the number of neurons with detectable Kiss1 mRNA in the ARC was significantly higher in *hpg* compared to WT mice (WT females, 47.5±8.3; *hpg* females, 99.7±20.7; P<0.05; WT males, 42.3±7.3; *hpg* males, 117.8±17.5 neurons/section; P<0.05). There was also an associated increase in the silver grain signal density in both male and female *hpg* mouse Kiss1 mRNA-positive neurons as measured by the cellular brightness of each neuron (P<0.05) **(** **Fig. 5C** **)**. These findings indicated that the clusters of enlarged kisspeptin-immunoreactive neurons in the ARC of *hpg* females and males represent neurons undergoing robust *Kiss1* expression.

### Pubertal Increases in *hpg* ARC Kiss1 mRNA are Not Detected in WT Mice

**Figure 6:**
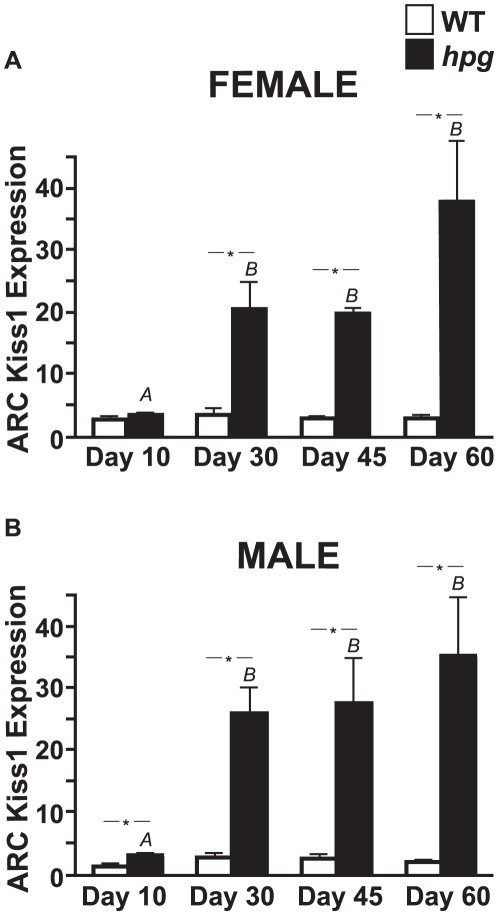
Developmental expression of *Kiss1* in the ARC of male and female *hpg* and WT mice. Quantitative analysis of Kiss1 mRNA levels in the ARC measured by qRT-PCR of WT and *hpg* females (**A**) and males (**B**) across postnatal development. Values are mean ± SEM. WT, *white bars*; *hpg*, *black bars.* Different letters denote significant differences (ANOVA, Neuman-Keuls post hoc). *, denotes significant differences between genotypes, P<0.05.

To more accurately measure changes in ARC *Kiss1* expression during development, total RNA was extracted from the ARC and used to measure Kiss1 mRNA levels by qRT-PCR in male and female WT and *hpg* mice at ages 10, 30, 45 and 60 days. Similar to the ICC and ISH results, and in contrast to the results for the WT AVPV, there was no sexual dimorphism in ARC Kiss1 mRNA levels for either genotype **(** **Fig. 6A, B** **)**. There were no significant changes in ARC Kiss1 mRNA levels across maturation in either WT females or males (P>0.05; ANOVA). However, *Kiss1* expression in the ARC of both *hpg* females and males increased significantly after day 10 and remained elevated through maturation (P<0.05; ANOVA, Neuman-Keuls post-hoc) **(** **Fig. 6A, B** **)**. The differences in Kiss1 mRNA levels between *hpg* and age-matched WT females and males were found to be highly significant by two-way ANOVA (P<0.0001, both female and male) from day 30 onward (P<0.05; Bonferroni post-hoc).

### Adult *hpg* AVPV Kisspeptin Neurons are Refractory to Estradiol Positive Stimulation

**Figure 7:**
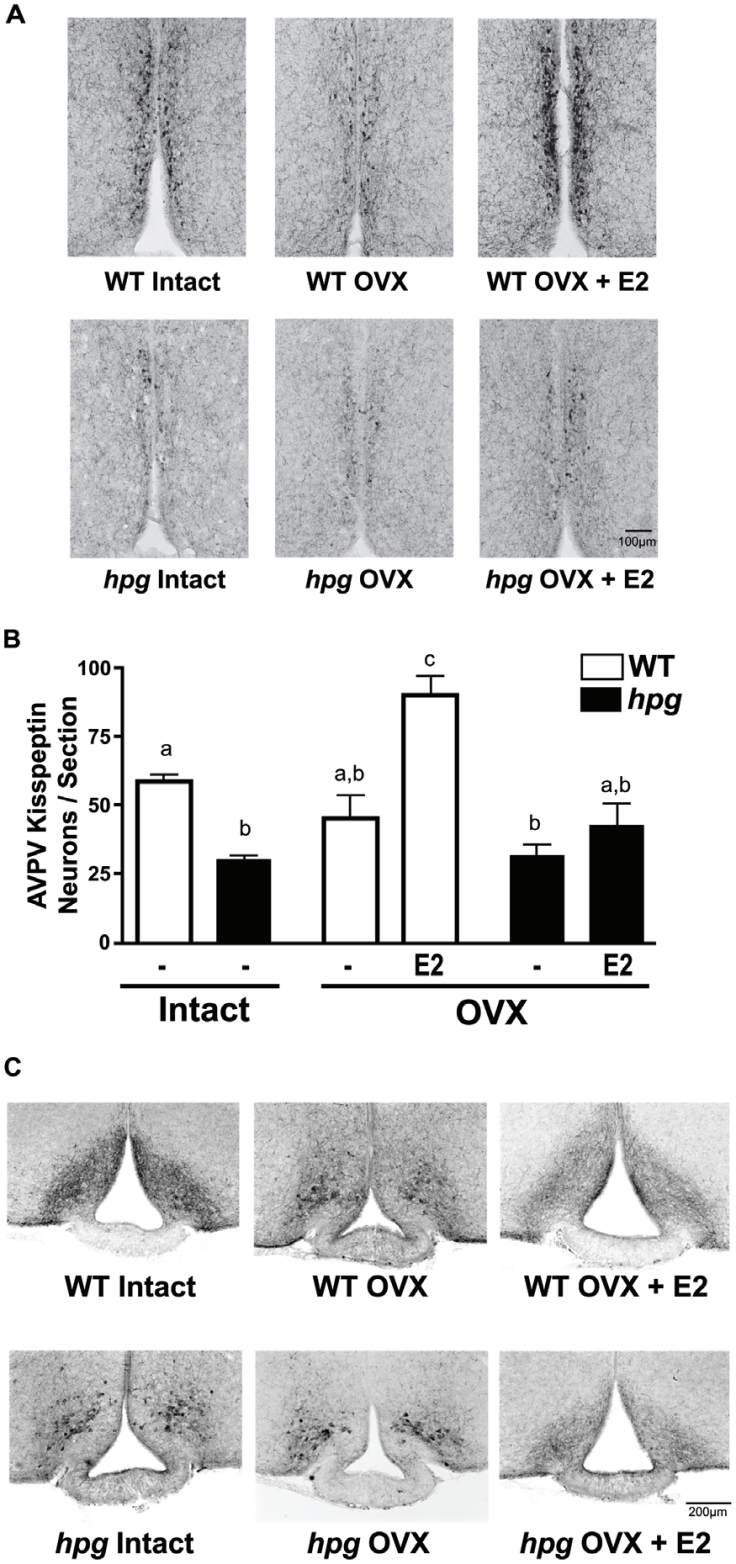
Effects of estradiol (E2) on *hpg* AVPV and ARC kisspeptin immunostaining patterns. **A**) Representative images of AVPV kisspeptin immunostaining patterns from gonad intact or ovariectomized (OVX) 60 day old WT and *hpg* females treated with sham or E2-containing Silastic capsules for 7 days. Scale bar  = 100 µm. **B**) AVPV kisspeptin-immunoreactive neurons from each group in (A) were counted and compared. Values are mean ± SEM. Different letters denote significant differences (ANOVA, Neuman-Keuls post hoc), P<0.05. **C)** Representative images of kisspeptin immunostaining patterns in the ARC of intact diestrus or OVX female WT or *hpg* mice, treated with sham or E2-containing Silastic capsules for 7 days. Scale bar  = 200 µm.

To test the potential capability of AVPV neurons in *hpg* mice to respond positively to exogenous estradiol (E2) with an increase in kisspeptin, as observed in WT mice [9], and to attempt to restore the numbers of AVPV kisspeptin-immunoreactive neurons to levels observed in WT mice, female *hpg* mice were ovariectomized (OVX) at 60 days of age and treated with E2-containing Silastic capsules for one week. WT mice were included as positive controls, and sham-treated groups were included as negative controls. E2 levels were significantly higher in mice receiving E2-filled capsules compared to control OVX mice receiving empty capsules, and uterine weights increased significantly in both the E2-treated OVX WT and *hpg* females (data not shown). Though ovariectomy resulted in a dramatic reduction in kisspeptin staining in the WT AVPV (**Fig. 7A**), the actual number of individual neurons with kisspeptin staining did not significantly decrease compared to the number in intact WT (intact diestrus WT, 62.7±4.4 neurons/section; OVX WT, 51.0±8.3 neurons/section; P>0.05; Neuman-Keuls post-hoc) **(** **Fig. 7B** **)**, This difference from previous observations using ISH following ovariectomy [9], [14] may reflect differences in the changes in kisspeptin immunoreactivity compared to the measurement of Kiss1 mRNA. Intact *hpg* mice had significantly fewer kisspeptin-positive neurons (30.4±1.7 neurons/section) than intact WT mice (P<0.05; Neuman-Keuls post-hoc), and OVX did not result in any further decrease in the *hpg* AVPV kisspeptin-positive neuronal population (32.0±4.1 neurons/section) **(** **Fig. 7A, B** **)**. E2 treatment of OVX WT females for 7 days significantly increased the number of kisspeptin-immunoreactive neurons in the AVPV, by 67% (85.0±7.0 neurons/section; P<0.01; Neuman-Keuls post-hoc), as expected, and exceeded the number of kisspeptin-immunoreactive neurons present in the AVPV of intact diestrus females. In contrast, E2 failed to significantly increase the number of AVPV kisspeptin neurons in OVX *hpg* females (43.4±8.0 neurons/section), resulting in no differences compared to untreated OVX or intact *hpg* mice (P>0.05; Neuman-Keuls post-hoc).

### Responsiveness of *hpg* ARC Kisspeptin Neurons to Estradiol Remains Intact

The functional response of kisspeptin neurons in the ARC to exogenous E2 was also examined by monitoring the changes of kisspeptin immunostaining patterns surrounding the median eminence. Adult WT diestrus female mice demonstrated dense kisspeptin neuropil immunostaining without clearly identifiable kisspeptin immunoreactive cell bodies **(** **Fig. 7C** **)**. One week following OVX, the WT ARC kisspeptin staining pattern was remarkably similar to that observed in the ARC of *hpg* mice, that is, darkly stained cell bodies with reductions in fiber immunostaining patterns **(** **Fig. 7C** **)**. With E2 replacement, the kisspeptin immunostaining pattern was restored to that observed in the intact WT females **(** **Fig. 7C** **).** In intact *hpg* mice, as above, the ARC kisspeptin immunostaining pattern again showed dark somatic staining with reductions in fiber staining, with no changes in pattern with OVX **(** **Fig. 7C** **)**. However, E2 treatment of OVX *hpg* mice rescued the kisspeptin immunostaining pattern to that observed in intact WT mice. These results suggested that a functional feedback response to E2 in the ARC had developed properly and was intact in *hpg* mice **(** **Fig. 7C** **)**.

## Discussion

It is becoming widely recognized that kisspeptin is essential for the initiation of reproductive function. However, the upstream regulators that drive kisspeptin expression have remained obscure, largely due to differences among species, sex and gonadal status during development [20]. Recent studies have affirmed the importance of a developmental role of sex steroids and underscored the need for focused efforts to further elucidate the influence of reproductive hormones on the kisspeptinergic system [20]. The present study addresses this matter further by investigating the maturation of both female and male hypothalamic kisspeptin in a background of GnRH deficiency. Markers associated with the occurrence of puberty, such as increases in gonadotropins and changes in genitalia, do not occur in *hpg* mice; accordingly, we have focused on the changes in kisspeptin expression as a key central neuroendocrine event to mark pubertal onset in rodents [18]. Our findings indicate that at the ages of normal pubertal maturation, increased kisspeptin is clearly detected in the *hpg* females and males, despite the absence of any appreciable exposure to gonadal sex steroid hormones. Thus, physiologic cues have remained sufficient for the induction of kisspeptin despite the hypogonadal phenotype.

By using a combination of ICC, ISH and qRT-PCR, changes in kisspeptin/Kiss1 highlighted the impact of the gonadal status of the *hpg* model. We have also identified disparities between Kiss1 mRNA and kisspeptin protein levels across development, suggesting differences between gene expression, peptide stability and intracellular distribution of kisspeptin. Also, as previously shown, we provide additional clear evidence of the role of gonadal sex steroids in sexual differentiation of the kisspeptinergic system, as these hormones are required for the feminization and masculinization of AVPV kisspeptin neurons [12], [21], [22], [23]. Further, the absence of positive feedback responsiveness of AVPV kisspeptin neurons to estrogen in *hpg* females suggests a critical dependence upon developmental gonadal hormone exposure for the establishment of a neural mechanism essential for the preovulatory LH surge. In contrast, the *hpg* ARC kisspeptin neurons displayed robust increases in developmental Kiss1 expression in the absence of negative gonadal sex steroid feedback, suggestive of an intact upstream central mechanism driving puberty onset in both sexes. Finally, we observed that the kisspeptin immunoreactive staining pattern in the ARC is dependent upon gonadal status and is reversible, as kisspeptin staining in the ARC of *hpg* mice treated with E2 is converted to a WT pattern. Taken together, these findings disclose new concepts in both the organization and developmental physiology of kisspeptin neurons.

In the AVPV, *Kiss1* expression increased only in WT females, not in WT males or in *hpg* mice. The increase in WT females likely reflected positive feedback stimulation by gonadal sex steroids following pubertal activation. Similar increases were reported by Takase, *et al*. in intact female rats between P21 and P26 [23]. The increase in AVPV *Kiss1* expression in pubertal WT females may contribute to the activation of the reproductive axis and is a potential factor contributing to the earlier age of puberty in females than in males, since this increase in Kiss1 mRNA was not significant in males by either ISH or qRT-PCR. Despite the absence of increased AVPV Kiss1 mRNA in *hpg* mice, there was a detectable increase in immunoreactive AVPV kisspeptin neurons in both *hpg* females and males at P30. This interesting discrepancy between mRNA levels and detectable immunoreactivity may reflect differences in the stability of Kiss1 mRNA and kisspeptin protein, or may represent accumulation of kisspeptin intracellularly due to the absence of a stimulus for release. Such differences between gene expression and peptide levels have been reported for other neuropeptides, such as CART responses to E2 [24]. The increased abundance of peptide not accompanied by a corresponding increase in steady-state levels of mRNA was also reported for hypothalamic EGFR expression in rats entering puberty [25].

In the AVPV, the development of kisspeptin neurons *requires reproductive hormones* in both females and males to establish the sexually dimorphic pattern normally evident in WT mice. In the *hpg* mouse model, the sexual modification of the AVPV kisspeptin neuronal population does not occur. The disparity between the WT females and males in the number of kisspeptin-immunoreactive neurons and between *Kiss1* expression levels in the AVPV was apparent by 30 days of age, as reported by Clarkson and Herbison with ICC [7], and at P18 by Kaufmann *et al*. with ISH [22]. In contrast, the AVPV population was the same size in both male and female *hpg* mice and intermediate in size to those of WT females and males at P30. The lack of sexual dimorphism in AVPV kisspeptin neurons in *hpg* mice is best explained by the escape of *hpg* males from the masculinizing effects of sex steroid hormones known to occur from perinatal testosterone (T) exposure. This was experimentally shown in perinatal female rats treated with T, which reduced AVPV neuronal *Kiss1* expression and population size to that of males [12], [21]. This is consistent with our findings of more AVPV kisspeptin-immunoreactive neurons in *hpg* over WT males. On the other hand, Kiss1 mRNA levels in *hpg* males by both ISH and qRT-PCT was not greater than WT males, a difference best explained by reduced activity of *Kiss1* transcription expected in the *hpg* males than in the gonad intact WT males [10]. Thus, in this case, the basal level of kisspeptin immunoreactivity illustrated the result of reproductive hormones to organize the AVPV kisspeptin population rather than *Kiss1* gene expression.

In postpubertal *hpg* females, kisspeptin immunoreactivity was increased in AVPV neurons, but only to half the level found in WT diestrus female mice at the same ages. This difference would be consistent with the absence of positive feedback effects by estrogen on *Kiss1* expression in the AVPV of *hpg* females. Exogenous estrogen increases *Kiss1* expression and the number of detectable kisspeptin-immunoreactive neurons in the AVPV, which suggests the pubertal activity of the gonads in WT females likely underlies the greater developmental increases above that found in *hpg* females, and *hpg* males as well [9], [26]. Similarly, kisspeptin/Kiss1 expression in the AVPV was described previously as reduced in the AVPV in the absence of E2 signaling of aromatase-deficient (ArKO) female mice and by ISH in female ERαKO mice [9], [17], [27]. By qRT-PCR, the reduction of Kiss1 mRNA in the *hpg* female AVPV was more dramatic than the *hpg* kisspeptin-immunoreactive neurons counted with ICC. Detection of peptide and mRNA together supported the requirement of reproductive hormones for the normal establishment and maintenance of the female AVPV kisspeptin neuronal population.

If the reduction of immunoreactive kisspeptin in *hpg* females results from the lack of E2 positive feedback given their absence of gonadal maturation, then we might predict that exogenous estrogen would rescue the number of immunodetectable kisspeptin neurons to levels in WT females. Functional studies with E2 administration (which resulted in measurable increases of circulating E2 levels and evidence of effects in the ARC) failed to increase the number of immunodetectable kisspeptin neurons, and levels were unable to be restored to those observed in WT. This suggests a critical developmental requirement of reproductive hormones for female AVPV kisspeptin neurons to effectively mount a positive feedback response to gonadal sex steroids, a finding that agrees with the interpretation put forth by Gonzalez-Martinez, *et al*. and Bakker, *et al*. [27], [28]. Physiologically, this effect may be important to sensitize kisspeptin neurons in the AVPV to increasing estrogen at the onset of puberty, and may be critical for maturation of the positive feedback mechanism necessary to mediate a GnRH/LH ovulatory surge in adult females. An alternative explanation of the failure of E2 treatment to increase the number of kisspeptin-immunoreactive neurons in the AVPV of *hpg* female mice that has not been ruled out is a need for hormonal priming, possibly by progesterone [29], [30].

Distinct from the AVPV, earlier studies have demonstrated the lack of sex differences in the ARC kisspeptin neurons in rodents [7], [12], [22], [31]. Our findings in the ARC are in agreement with these previous reports. In addition, we find substantial and sustained increases in ARC *Kiss1* expression at pubertal ages in both *hpg* females and males in the absence of reproductive hormones (**Fig. 5**). Therefore, the hormone-independent increase in *hpg* kisspeptin expression in the region of the ARC nucleus is consistent with studies that have classically demonstrated that the central mechanisms regulating the onset of puberty are not gonad dependent [32], [33], [34]. It should be noted that these observations do not rule out additional developmental changes in the sensitivity of ARC kisspeptin neurons to steroid negative feedback, as reported by others [23], [35], [36].

In the absence of gonadal sex steroid inhibition, ARC *Kiss1* expression is markedly increased by P30 in both female and male *hpg* mice. This pubertal increase in kisspeptin would normally initiate increased GnRH release and thereby lead to activation of the gonads; however, in WT mice, this increase in ARC Kiss1 mRNA is not appreciated. We propose that in intact WT mice, increases in ARC *Kiss1* expression are counteracted by negative feedback effects of increasing sex steroid hormones that occur as a result of the peripubertal activation of gonadal function. This would effectively clamp *Kiss1* expression at a constant level, reinforcing the existence of a sensitive homeostatic regulatory mechanism. Indeed, the absence of pubertal decreases in Kiss1 mRNA levels in WT mice despite the negative feedback effects of rising sex steroid hormone levels suggests underlying stimulatory inputs are also active. We take these findings to indicate that *Kiss1* expression is tightly regulated across the onset of puberty by both activational and repressive mechanisms.

Our findings differ from a developmental increase reported in the ARC Kiss1 of intact rats by Takase, *et al*. [23]. In this report, the role of E2 in peripubertal changes in kisspeptin expression was analyzed in the ARC and AVPV under controlled E2 conditions to reveal an increase in pubertal ARC Kiss1 mRNA expression and kisspeptin immunoreactivity in intact female rats between P21 and P26 [23]. Increases in ARC Kiss1 mRNA was not found in our study in intact mice between P10 and P30 and may represent differences in species, time points studied, or methodology in qRT-PCR expression normalization; e.g. in our protocol we controlled for developmental changes in ARC size. There were also differences between approaches to statistical analyses. Further, in contrast to our measurements in *hpg* mice, Takase, *et al*., did not observe increases in Kiss1 mRNA between P21 and adult OVX female rats, possibly because levels were already elevated in the ARC by P21. Nonetheless, our findings agree with the Takase, *et al*. conclusion that pubertal increases in Kiss1/kisspeptin expression in the ARC and in the AVPV may cooperatively contribute to female puberty [23].

Based on earlier studies that described ARC kisspeptin neurons as mediators of sex steroid negative feedback [9], [10], it was predicted that ARC *Kiss1* expression would be increased in *hpg* mice. Our observations with qRT-PCR were consistent with this regulatory role of the ARC kisspeptin neurons. Immunostaining of the peptide distribution pattern of kisspeptin in the ARC suggested additional differences, also dependent upon the gonadal status of mice, which altered the cellular distribution of kisspeptin. Kisspeptin fiber immunostaining in the WT ARC could not easily be quantitated, and contrasted with the dark cellular staining observed in *hpg* mice. Utilizing ISH and qRT-PCR confirmed that *hpg* mice had robustly elevated *Kiss1* expression in striking contrast to kisspeptin immunostaining, which could easily be misinterpreted as reduced kisspeptin immunoreactivity in the *hpg* ARC. This difference in the kisspeptin cellular distribution pattern may reflect rapid release of the neuropeptide from axon terminals, or alternatively altered axonal transport to result in a reduction of axonal kisspeptin content. If correct, this cellular pattern of ARC kisspeptin staining would be expected under other hypogonadal conditions. Indeed, we have observed this pattern in OVX WT mice (**Fig. 7C**). A similar pattern was recognized in published images of ARC kisspeptin staining of homozygous ArKO female mice [17] and also observed in *Kiss1R* ^−/−^ mice (data not shown). Moreover, the immunostaining pattern in the *hpg* ARC is reminiscent of the description of hypertrophied kisspeptin neurons reported by Rance *et al*. in the ARC nucleus of postmenopausal human females also consistent with the conditions of low levels of circulating E2 [37], [38]. Interestingly, this staining pattern has also been reported for other ARC neuropeptides under similar conditions of changes in physiological neuroendocrine regulation [39], [40].

In functional tests of *hpg* kisspeptin neuron responsiveness, the distribution of kisspeptin immunoreactivity in the OVX+E2-treated *hpg* ARC was restored to the pattern found in WT mice. This contrasts the lack of response of *hpg* AVPV kisspeptin neurons to E2 replacement. This is important for several reasons. First, it demonstrated that fibers of kisspeptin-positive neurons in the ARC were intact and likely developed normally despite the absence of reproductive hormones. Second, this experiment illustrated the plasticity of kisspeptin distribution in axonal fibers; appearing as neuropil staining in the presence of E2, but under conditions of low E2, greater somatic staining with minimal kisspeptin immunoreactivity in neuronal fibers. Finally, the change from the dense cellular staining in *hpg* ARC, corresponding to elevated *Kiss1* gene expression, to a WT pattern of kisspeptin neuropil, suggests E2 restored kisspeptin distribution to a pattern found at low *Kiss1* expression. This response provides further support of an intact negative feedback mechanism in the ARC of *hpg* females, despite the lack of developmental reproductive hormone exposure.

The present study highlights that pubertal kisspeptin synthesis in ARC kisspeptin neurons proceeds in *hpg* mice independent of reproductive hormones. This study also indicates a critical dependence of AVPV kisspeptin neurons on reproductive hormones to distinguish female and male kisspeptin neuronal organization and function. The complex maturation process that gates the pubertal transition appears to advance through ARC kisspeptin synthesis that is initially detected in both female and male *hpg* mice at 30 days, an effect difficult to detect in the intact WT as a result of negative feedback effects of sex steroid hormones. AVPV kisspeptin neuronal maturation, in turn, is shaped either by perinatal masculinization or by developmental feminization by gonadal sex steroids. More specifically, the absence of developmental reproductive hormone exposure in *hpg* females restricted the AVPV kisspeptin neuronal population size and/or *Kiss1* expression at puberty. This finding suggests that reproductive hormone exposure may mediate ultimate kisspeptin levels in the adult female and subsequently affect the sensitivity of positive sex steroid feedback responses in the AVPV.

## Materials and Methods

### Animals

All experiments were approved by the Harvard Medical Area Standing Committee on Animals in the Harvard Medical School Center for Animal Resources and Comparative Medicine. Mice were maintained in a 12∶12 h light/dark cycle and fed standard rodent chow. Mice heterozygous for the *Gnrh1* gene deletion (CB17; *HPG*-*Gnrh1^hpg^*) were bred to generate homozygous (*hpg*), heterozygous, and wild-type (WT) mice. Genotypes were determined by standard PCR of genomic tail DNA performed with three primers: primer oIMR 903: 5′-TATGGCTTACAGTTCCAGCG; oIMR 904: 5′-AGGCTTGGAGAGCTGTAAGG and primer oIMR 905: 5′-GTTTCAGTGCATCCTCTCAGG (Jackson Laboratory, Bar Harbor, ME). In experimental studies, only male and female homozygous *hpg* or WT littermates aged 10 days (P10) to adulthood (P60) were used. Homozygous *hpg* mice were phenotypically hypogonadal, with micropenis or delayed or absent vaginal opening in adult males and females, respectively, but were similar in size to their WT counterparts. When intact female WT mice aged P45 and older were used, they were confirmed to be in the diestrus phase of the estrous cycle at the time of the study based on vaginal cytology.

### Kisspeptin Immunocytochemistry

Intact male and female *hpg* mice and WT littermates aged P10, P30, P45 and P60 (n = 4–6 per group) were killed by pentobarbital anesthetic overdose and intracardial perfusion with 30 ml of buffered 4% paraformaldehyde. Brains were removed and postfixed for 1–2 h in the same fixative and cryoprotected in 30% sucrose. Coronal cryosections (40 µm thickness) containing the AVPV and ARC were collected in 0.1 M phosphate-buffered saline (PBS) and alternating sections were processed for mouse kisspeptin immunocytochemistry (ICC). Floating sections were washed with 1% hydrogen peroxide in PBS containing 0.4% Triton ×100 (PBST) for 30 min to quench the endogenous peroxidase activity, rinsed five times with PBST, and incubated for 72 h at 4C in PBST containing rabbit anti-mKiSS(112–121)/m*Kiss1*0 antibody (#564, 1∶15,000; a gift from Dr. Alain Caraty, Univ. Tours, FR), 10% normal horse serum and 4% normal donkey serum. After primary antibody incubation, sections were washed with PBST and incubated with a biotinylated donkey-anti-rabbit IgG (1∶400; Jackson Laboratory, West Grove, PA) for 1 hour. Sections were washed, incubated with Elite ABC-peroxidase (Vector Labs, Burlingame, CA), and then incubated for 10 min in ImmPACT DAB substrate (Vector Labs) for visualization. Serial sections were mounted on microscope slides, dehydrated and mounted with coverslips with Permount (Sigma), and visualized and imaged with a conventional stereomicroscope equipped with a digital camera. Sample identification was coded and obscured to the analyst prior to cell counting. Individual kisspeptin neurons were counted from sections of the AVPV having the greatest total number of kisspeptin neurons. This method was utilized rather than counting neurons throughout the entire AVPV to overcome two issues: (1) kisspeptin neurons are found in a continuum along the ventricular regions of the third ventricle, making it difficult to clearly define boundaries of the AVPV/RP3V population of neurons, and (2) the size of the AVPV itself is sexually dimorphic, adding further difficulties to select comparable areas for counting. In the ARC, kisspeptin immunoreactivity was digitally imaged. For immunocytochemistry controls, brains from adult male and female homozygous *Kiss1^−/−^* mice (kindly provided by S. Seminara, Massachusetts General Hospital, Boston, MA)[5] were used to validate kisspeptin antibody specificity by staining sections of AVPV and ARC prepared as above and were devoid of positive kisspeptin immunoreactivity (data not shown).

### *In Situ* Hybridization

Pubertal WT and *hpg* male (P45) and female mice (P30; n = 4 per group) were killed by CO2 asphyxiation and brains were removed and rapidly frozen on dry ice. Coronal sections (20 µm) of the AVPV and ARC were cut on a cryostat, thaw-mounted onto SuperFrost Plus slides (VWR Scientific, West Chester, PA), and stored at −80 C. Antisense mouse Kiss1 riboprobe, corresponding to the published sequence of the *Kiss1* mouse gene (GenBank accession no. AF472576) spanning bases 76–486, was transcribed from linearized pAMP1 plasmid containing the mouse *Kiss1* insert (kindly provided by R. Steiner, University of Washington, Seattle, WA). The validation of this Kiss1 probe specificity was previously determined [41]. Radiolabeled probes were synthesized *in vitro* in a volume of 20 µl containing 50 µCi ^33^P-UTP (PerkinElmer Life Sciences, Boston, MA); 1.25 µg linearized DNA; 0.5 mM each ATP, CTP, and GTP; 40 U T7 polymerase; 1 µl RNase inhibitor; 1.25 µl 0.1 M DTT and 4 µl 5X transcription buffer. Residual DNA was digested with 4 µU DNase (Ambion, Austin, TX) and the DNase reaction was terminated by addition of 80 µl of STE buffer (100 mM NaCl, 20 mM Tris–HCl, pH 7.5, 10 mM EDTA). The riboprobe was separated from unincorporated nucleotides with RNeasy purification kit (Qiagen) and quantified in a scintillation counter. Radioactive *in situ* hybridization was performed similarly to a previously described protocol [42]. Briefly, frozen slides were thawed, fixed in 4% paraformaldehyde, then delipidated in chloroform, dehydrated in graded ethanols and acetylated in triethanolamine buffer. Radiolabeled, antisense riboprobe was denatured, diluted in hybridization solution at a concentration to yield 10^6^ cpm/ml and applied to slides (130 µl/slide). Slides were covered with glass coverslips and incubated in a humidified chamber at 65 C for 16 h. After hybridization, slides were treated with RNase (32 µg/ml), washed under conditions of increasing stringency, dehydrated and air dried. Slides were then dipped in Kodak NTB-3 liquid emulsion (Carestream Health, Rochester, NY). Slides were developed after 13 d exposure and mounted with coverslips. ISH slides were imaged with a digital camera-equipped Carl Zeiss Axioskop microscope with darkfield illumination (Carl Zeiss, Thornwood, NY), allowing identification of silver grain clusters corresponding to Kiss1 mRNA-expressing cells. Nikon NIS Elements software (Nikon Instruments, Melville, NY) was used to automatically identify and count individual Kiss1 mRNA-positive cells by isolation of silver-grain signals that met threshold criteria of size and intensity above uniformly normalized background. Nikon NIS Elements software (Nikon Instruments) was also used to estimate the signal strength of identified Kiss1-positive neurons/section by silver-grain signal estimation, by use of the measurement parameter deemed Sum Brightness (the calibrated brightness of all pixels per object) and averaged for all positive neurons.

### Kiss1 Real Time qRT-PCR

For real time qRT-PCR, intact male and female *hpg* mice and WT littermates aged P10, P30, P45 and P60 (n = 3–6 per group) were killed, brains were removed and coronal sections of brain tissue (1 mm thick) encompassing the regions containing either the ARC and AVPV were obtained with the aid of a 1 mm coronal brain matrix (Braintree Scientific, Braintree, MA). The AVPV and ARC were then isolated from each section under a dissection microscope with fine instruments by two bilateral parasagittal cuts, each 0.5 mm lateral to the midline, and one horizontal cut 1 mm dorsal of the ventral surface, yielding total tissue sizes of approximately 1 mm^3^ that were immediately frozen in liquid nitrogen and stored at −80 C. Large margins were taken to ensure that each tissue sample encompassed the entire kisspeptin neuronal population from either the AVPV or ARC from each animal. Total RNA was later extracted from the collected tissues (Trizol, Invitrogen, Carlsbad, CA) and resuspended in equal volumes of H_2_O. Total RNA concentration was carefully measured by UV spectrophotmetry and RNA integrity was confirmed by gel electrophoresis. We employed an accurate way of adjusting for size of the dissected material assumed to encompass the entire target kisspeptin population. To account for dilution of Kiss1 mRNA signal by non-target mRNA species in the tissue specimens, a sample-size normalization method was used that based sample size on the total RNA recovered from each brain region [43]. *Kiss1* expression was measured based on the amount of total RNA extracted from the tissue which is known to have a linear correlation (r^2^ = 0.99) between the quantity of starting tissue sample and the yield of purified RNA extracted by a Trizol method (Invitrogen, Carlsbad, CA). Proportional amounts of total RNA, representing 15% of recovered total RNA (range 0.25–5 µg), were reverse transcribed for each sample, using a standard reverse transcription kit with oligo dT primers according to manufacturer's instructions (Superscript III kit, Invitrogen). Quantitative real-time PCR was performed with an ABI 7300 Real-Time PCR System (Applied Biosystems, Foster City, CA) with the Taqman detection system for amplification using primer sequences specific for *Kiss1* (Id: MM00617576_M1, Applied Biosystems). Replicates (n = 2) were performed for each sample to obtain an average threshold cycle value (Ct). No Kiss1 amplification was detected from negative control samples including cerebellar and liver tissues from subsets of WT and *hpg* mouse samples, samples prepared without reverse transcriptase, or from water controls. Expression of *Kiss1* was calculated by mRNA quantitation for each sample relative to the mean Ct value from day 10 female WT AVPV samples as a benchmark by the ΔCt method and expressed as fold change from that benchmark. Experimental control for uniform cDNA synthesis and loading included amplification of mouse β-actin (Taqman Id: 4352933E; Applied Biosystems, Inc.) as a housekeeping gene from each sample that demonstrated proportional β-actin expression to the amount of total RNA as a control for any inherent variability of the reverse transcription reaction [44].

### Steroid Hormone Replacement

Bilateral OVX was performed on adult (P60) female WT and *hpg* mice (n = 4 per group) under pentobarbital anesthesia delivered intraperitoneally. Vasculature to the ovary was sutured and the incision was closed by suture and wound clips. Immediately after OVX, E2-filled capsules were implanted subcutaneously via a small incision at the base of the neck; wound clips were used to close the incision. For E2 implants, Silastic tubing (inner diameter 1.47 mm; outer diameter 1.95 mm; Dow Corning, Midland, MI) was cut to 9 mm; one end was sealed with silicone cement and allowed to cure overnight. The dose of crystalline E2 (Sigma, St. Louis, MO) was based on a previous study and involved packing Silastic tubing with 4 mm of an E2/cholesterol mix (1∶4) [10]. The day before surgery, implants were washed with two 10 min changes of 100% ethanol and then rinsed and stored in physiological saline (0.9% saline) overnight. All untreated animals received empty (sham) capsules. All mice were sacrificed seven days after capsule treatment and the brains were prepared for kisspeptin ICC of the AVPV and ARC nuclei and results analyzed as described earlier.

### Data analysis

Multiple comparisons were analyzed by ANOVA followed by the appropriate post-hoc analyses. Parametric statistics were used with data that satisfied Bartlett tests for equal variances. Otherwise, transformed or nonparametric statistics were used. Suitable results were analyzed by unpaired *t* test for the comparison of mean differences between two different genotypes of similar age. Data were analyzed using Prism statistics software (GraphPad Software, Inc. San Diego, CA) when possible. All data are presented as the mean ± SEM. Differences were considered significant when P<0.05.
